# TRIM47 overexpression is a poor prognostic factor and contributes to carcinogenesis in non-small cell lung carcinoma

**DOI:** 10.18632/oncotarget.15188

**Published:** 2017-02-08

**Authors:** Yudong Han, Haiying Tian, Pei Chen, Qiang Lin

**Affiliations:** ^1^ Department of Thoracic Surgery, Shanghai General Hospital, Shanghai Jiao Tong University School of Medicine, Shanghai, China; ^2^ Renji Hospital, Shanghai Jiao Tong University School of Medicine, Shanghai, China

**Keywords:** TRIM47, NSCLC, cell cycle, P53, EMT

## Abstract

Non-small cell lung carcinoma (NSCLC) is the most common malignancy with the highest morbidity and mortality. In this study, we found that tripartite motif containing 47 (TRIM47) expression level was higher in tumor tissues than in normal adjacent tissues. Overexpression of TRIM47 closely correlated with poor prognosis in patients with NSCLC. Multivariate Cox regression analyses showed that TRIM47 overexpression could be considered an independent prognostic factor for NSCLC. TRIM47 depletion significantly inhibited cell proliferation and induced G1phase arrest in A549 and H358 cell lines. Moreover, TRIM47 silencing remarkably inhibited cell migration, cell invasion, and tumorigenicity in nude mice. Gene set enrichment analysis (GSEA) revealed that cancer-related process and pathways, including p53-cell cycle and NFκB-epithelial mesenchymal transition (EMT) pathway, were significantly correlated with TRIM47 expression. Real-time PCR and Western blot analysis revealed that TRIM47 exerts an inhibitory effect on p53 and an facilitatory effect on NF-κB, thereby promoting tumor proliferation and metastasis. Taken together, TRIM47 acts as a tumor oncogene in NSCLC. Our data provide insight into the possible biological mechanism of TRIM47 in the progression of NSCLC and highlight its usefulness as a potential therapeutic target.

## INTRODUCTION

Lung cancer is a primary cause of cancer-related morbidity and mortality worldwide, accounting for nearly one-fifth of cancer deaths due to invasion, metastases and drug resistance [1, 2]. NSCLC constitutes nearly 80% of lung cancer related deaths [3]. Currently available treatment strategies for NSCLC, including surgery, radiotherapy, and chemotherapy, have resulted in significant benefits but remain generally unsuccessful [4]. Despite incremental advancements in molecular therapeutics for the treatment of NSCLC, such as epithelial growth factor receptor-targeting therapies, patient survival has not significantly improved [5, 6]. Identification of the molecular mechanism of carcinogenesis and metastasis will facilitate the development of new therapeutic targets and anti-lung cancer strategies.

The TRIM family proteins are evolutionarily conserved proteins, composed of a ring finger domain, one or two B-box domains, and an associated coiled-coil domain in the amino-terminal region [7]. TRIM proteins are involved in a wide array of cellular processes, including cell proliferation [8], differentiation [9], apoptosis [10], cell cycle regulation, development [11], carcinogenesis [12], inflammation [13] and innate immunity [14]. Most TRIM proteins confer ubiquitin E3 ligase activity and promote post-translational modifications, including mediating ubiquitination events [15]. Several studies reported that TRIM proteins also act as oncogenes or tumor suppressors in several cancers. For instance, TRIM28 appears to be upregulated in lung cancer [16] and breast cancer [17]. Upregulation of TRIM59 is associated with poor prognosis in gastric cancer [18] and lung cancer [19]. Recently, Tetsuya et al. reported that high levels of TRIM47 expression were found to be a strong prognostic factor in prostate cancer [20]. However, little is known about the expression pattern and biological functions of TRIM47 in NSCLC.

In our current study, we explored the expression level, biological functions and potential molecular mechanisms of TRIM47 in NSCLC. TRIM47 mRNA was significantly overexpressed in tumor tissues compared to normal adjacent tissues. Then, we found that TRIM47 silencing inhibited cell proliferation, migration, invasion and xenograft tumor growth. Furthermore, gene set enrichment analysis (GSEA) on the RNA-sequencing data from The Cancer Genome Atlas (TCGA) indicated that high expression of TRIM47 was related with p53-cell cycle and NFκB-EMT signaling pathways, which were further identified in NSCLC cells. Collectively, our data suggest that TRIM47 is a potent prognostic factor and a potential target for the treatment of NSCLC.

## RESULTS

### TRIM47 gene up-regulation associated with poor survival in NSCLCs

**Figure 1:**
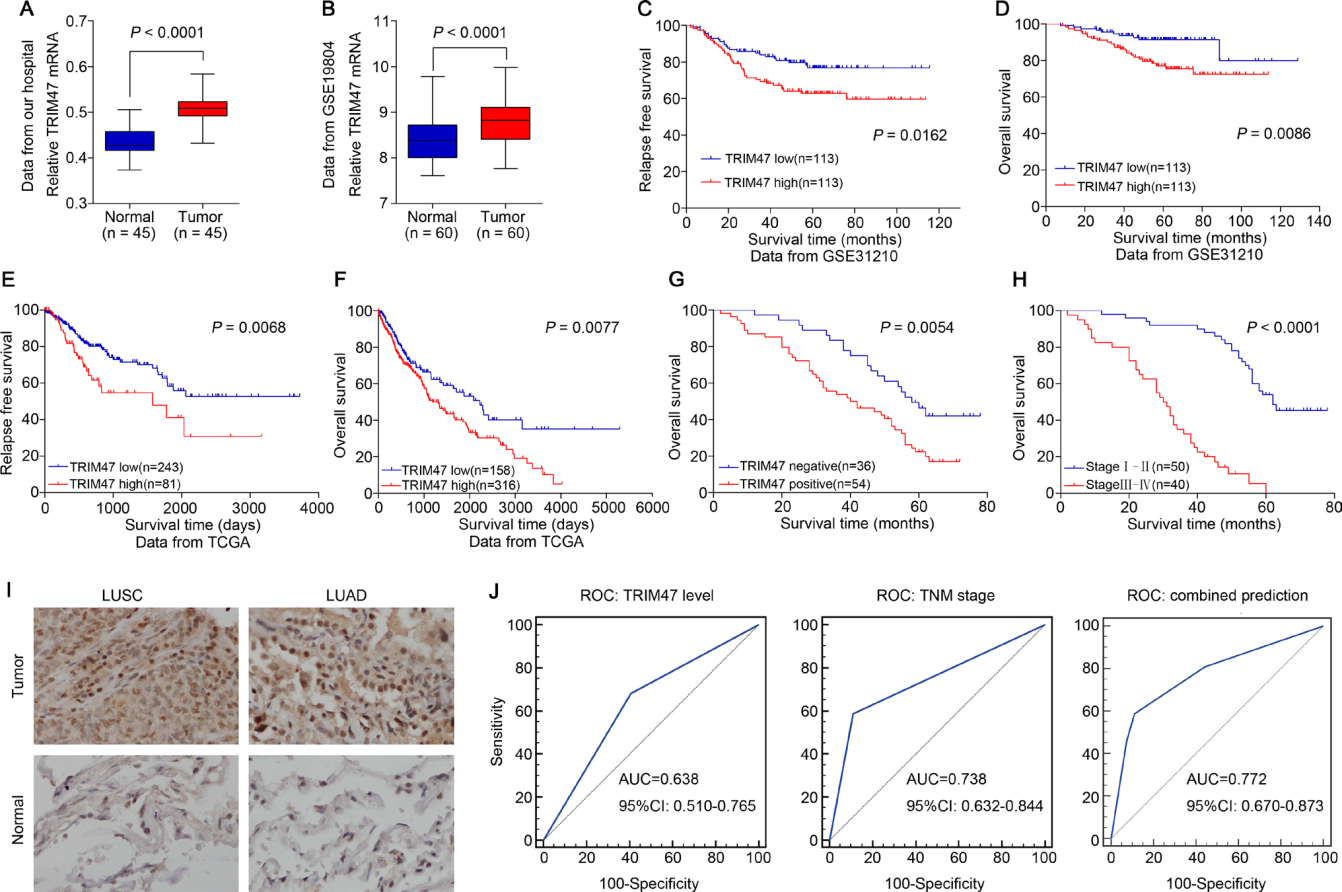
TRIM47 overexpression was associated with poor prognosis in NSCLC (**A**) The expression level of TRIM47 mRNA was higher in tumor tissues compared with normal adjacent tissues (*P* < 0.0001). (**B**) TRIM47 expression was significantly increased in NSCLC tumor tissues when compared with normal tissues from a GEO dataset (*P* < 0.0001). (**C**, **D**) Survival analysis of early stage NSCLC patients from a GEO dataset (GSE31210). (**E**, **F**) Kaplan-Meier survival analysis of NSCLC patients from TCGA dataset. (**G**) Kaplan-Meier survival analysis of NSCLC patients from the Department of Thoracic Surgery, Shanghai General Hospital (*P* = 0.0054). (**H**) Kaplan-Meier survival analysis of NSCLC patients with different TNM stage. (**I**) Immunohistochemical staining of TRIM47 in tumor tissues and normal adjacent tissues (LUSC: lung squamous cell carcinoma; LUAD: lung adenocarcinoma). (**J**) The receiver operating characteristic (ROC) curves for predicting patient survival using TRIM47 level, TNM stage or a combination of two factors. The area under curve (AUC) and the corresponding 95% CI are shown in the plots.

We first investigated TRIM47 expression level in 45 NSCLC tissues and normal adjacent tissues to determine its significance in NSCLC by real-time PCR. As shown in Figure 1A, TRIM47 mRNA levels were significantly increased in tumor tissues compared with normal adjacent tissues. Additionally, several multiple microarray datasets of NSCLC from TCGA and Gene Expression Omnibus (GEO) were analyzed. The GEO dataset (GSE19804, Figure 1B) demonstrated that TRIM47 expression level was significantly higher in NSCLC tissues than in normal tissues, which was consistent with our data. The GEO dataset (GSE31210, Figure 1C, 1D) and the TCGA dataset (Figure 1E, 1F) illustrated that higher TRIM47 expression predicted poor relapse-free survival and overall survival in early stage and advanced stage NSCLC, respectively. The above data suggested TRIM47 is critical during carcinogenesis and malignant progression of NSCLC.

Next, by analyzing the immunohistochemical results, we found that TRIM47 expression in the NSCLC samples was negative in 36 cases and positive in 54 cases. Based on the statistical results, TRIM47 expression was notably less prevalent in the normal adjacent tissues than in the NSCLC tissues (Figure 1I). Our data also illustrated that higher TRIM47 expression predicted poor overall survival (*P* = 0.0054, Figure 1G). Both the stratification by TRIM47 level and the widely used TNM staging (*P* < 0.0001, Figure 1H) displayed high prognostic significance. To evaluate the potential capability of TRIM47 as a diagnostic biomarker for the prediction of patient survival, receiver operating characteristic (ROC) curves were conducted using TNM stage, TRIM47 level, or a combination of both (Figure 1J). The area under the curve (AUC) of the TNM stage-based model and the TRIM47-based prediction was 0.738 and 0.638, respectively, and the combination of both factors yielded the highest AUC value (0.772).

Table 1 summarizes the association between TRIM47 expression and various clinicopathological parameters in 90 NSCLC patients. TRIM47 expression was correlated with tumor differentiation (*P* = 0.011), TNM stage (*P* = 0.002), lymph node metastasis (*P* = 0.003), and tumor size (*P* = 0.016). We got the same results on TRIM47 mRNA level in 45 NSCLC patients (Supplementary Table 2). Multivariate Cox regression analyses showed that along with TNM stage and lymph node metastasis, overexpression of TRIM47 (*P* = 0.017) could be considered an independent prognostic factor for NSCLC patients (Supplementary Table 1).

**Table 1:** Relationship between TRIM47 expression and clinicopathological parameters in NSCLC patients

Variables	No. of patients	TRIM47 expression		*P*
		Positive	Negative	
Age				0.577
< 58	28	18	10	
≥ 58	62	36	26	
Gender				0.928
Male	58	35	23	
Female	32	19	13	
Histological type				0.794
Squamous cell carcinoma	39	24	15	
Adenocarcinoma	51	30	21	
Tumor differentiation				0.011*
Well	53	26	27	
Poor	37	28	9	
TNM stage				0.002**
I + II	50	23	27	
III + IV	40	31	9	
Lymph node metastasis				0.003**
No	45	20	25	
Yes	45	34	11	
Tumor size				0.016*
< 3 cm	41	19	22	
≥ 3 cm	49	35	14	

Note: *P* values are from chi-square test and were significant at < 0.05. *P < 0.05, **P < 0.01.

### Silencing of TRIM47 inhibited cell proliferation and induced G1 phase arrest

**Figure 2:**
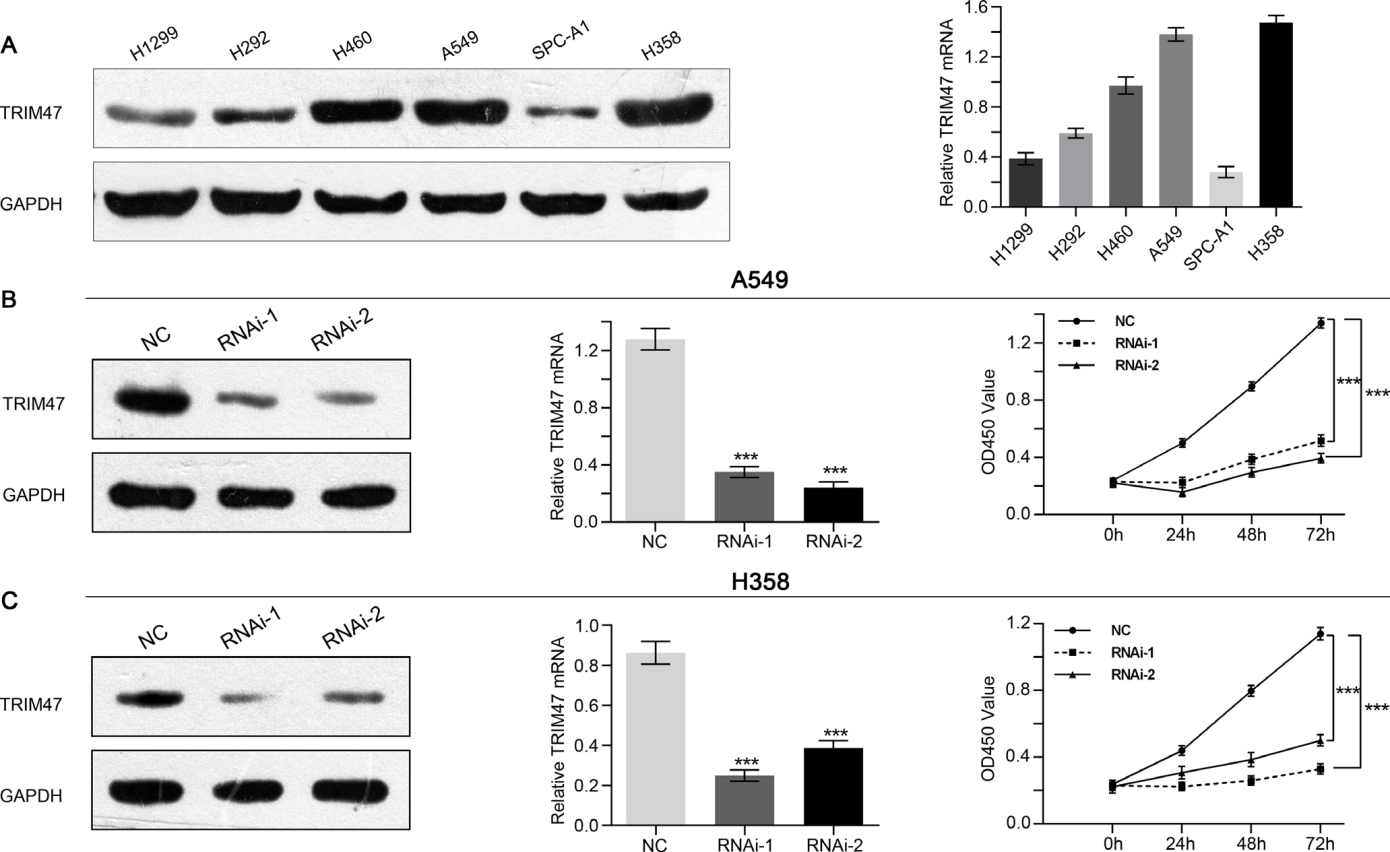
Depletion of TRIM47 inhibited the proliferation of NSCLC cells (**A**) TRIM47 expression level in six NSCLC cell lines was analyzed by real-time PCR (right panel) and Western blot (left panel). Data were based on at least three independent experiments. (**B**, **C**) Expression of TRIM47 in A549 and H358 cells was analyzed by Western blot (left panel) and real-time PCR (middle panel). Cell proliferation (right panel) was detected 24, 48 and 72 hours after infection in A549 and H358 cells. Data were based on at least three independent experiments and shown as the mean ± SD (***P < 0.001). NC: scrambled shRNA-infected cells; RNAi-1, RNAi-2: TRIM47-shRNA-infected cells.

We next estimated the expression level of TRIM47 in six NSCLC cell lines (A549, H460, H1299, SPC-A1, H292 and H358) by Western blot and real-time PCR. As shown in Figure 2A, two cell lines, A549 and H358, showed higher TRIM47 protein and mRNA expression and were chosen for further study. A non-specific scramble shRNA sequence (NC) and two shRNA sequences targeting TRIM47 were cloned into a lentiviral vector, and corresponding lentiviruses were produced to infect A549 and H358 cells. TRIM47 expression in A549 and H358 cells was efficiently suppressed by the two shRNA viruses (Figure 2B, 2C).

A CCK-8 assay was used to evaluate the effects of TRIM47 silencing on the proliferation of NSCLC cells. The proliferation was significantly suppressed by shRNA-TRIM47 compared with the NC group (Figure 2B, 2C). These data indicate that TRIM47 promotes proliferation in A549 and H358 cells.

**Figure 3:**
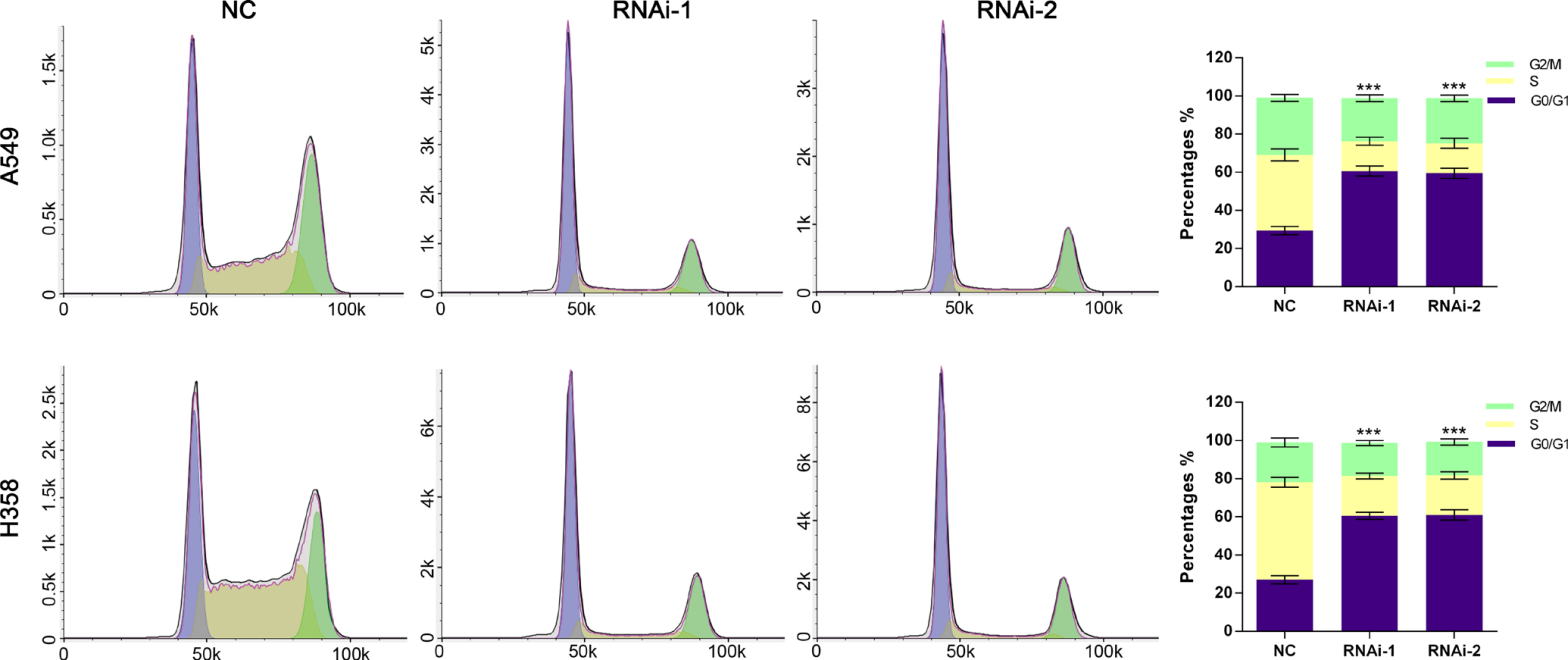
Silencing of TRIM47 induced G0/G1 arrest in NSCLC cells Cell cycle profile was analyzed using flow cytometry. Data were based on at least three independent experiments and are shown as the mean ± S.D (***P < 0.001).

PI staining and flow cytometry analysis were used to evaluate the effects of TRIM47 knockdown on the cell cycle. TRIM47-shRNA infection caused a significant increase of G0/G1 phase cells and a decrease of S and G2/M phase cells compared with the NC group (Figure 3).

### Silencing of TRIM47 inhibited the metastasis of NSCLC cells

**Figure 4:**
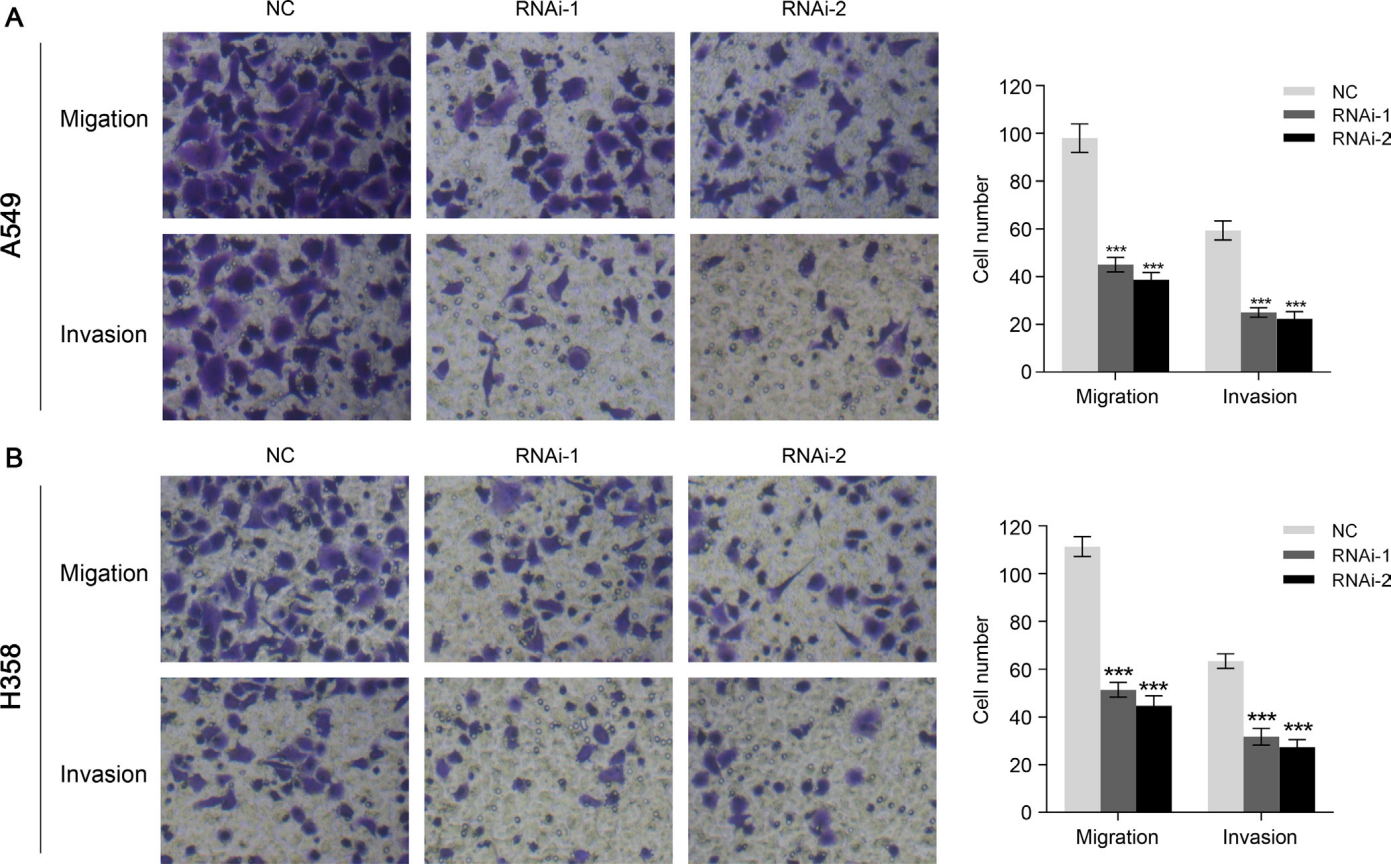
TRIM47 depletion inhibited cell migration and invasion in NSCLC A549 (**A**) and H358 (**B**) cells were infected with indicated virus. Knockdown of TRIM47 inhibited cell migration and invasion (***P < 0.001).

The migration and invasion ability of NSCLC cells were assessed to investigate the function of TRIM47 in the metastasis of cancer cells. In sharp contrast to control cells, suppressing TRIM47 expression showed remarkable reduced migration and invasion ability (Figure 4). Compared with the numbers of control cells that migrated to the lower side of the transwell membrane, TRIM47 knockdown cells suffered significantly inhibited motility. These data indicated a role of TRIM47 in the promotion of NSCLC metastasis.

### Knockdown of TRIM47 suppressed tumorigenicity of NSCLC cells in nude mice

**Figure 5:**
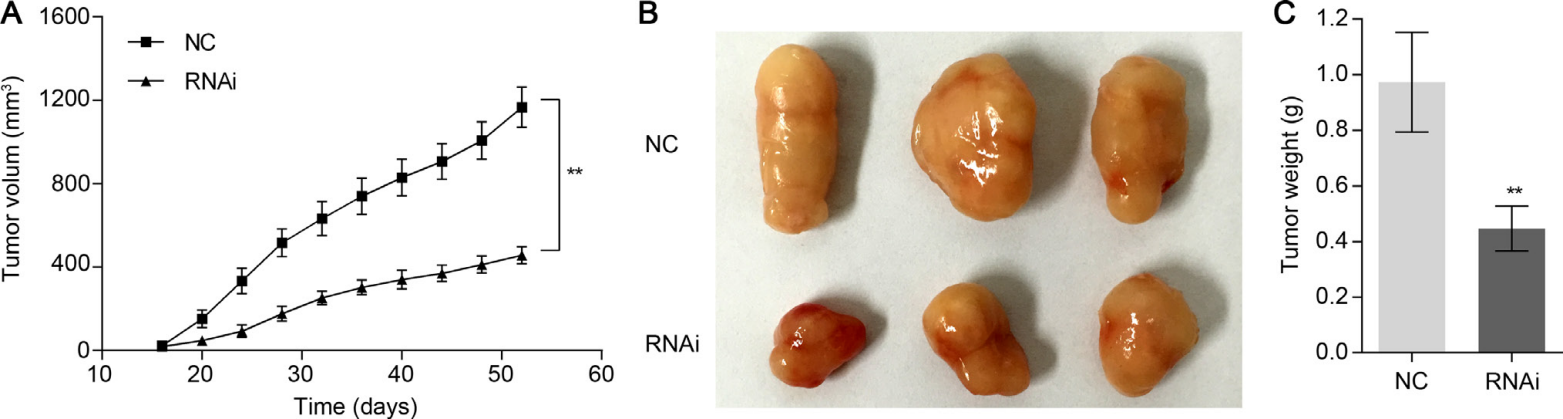
Knockdown of TRIM47 in NSCLC cells reduced tumor growth *in vivo* Knockdown of TRIM47 in A549 cells significantly inhibited tumor growth in nude mice xenograft model. (**A**) Tumor growth was significantly slower in mice injected with TRIM47 depletion cells (**P < 0.01). (**B**, **C**) Mice were sacrificed and tumors were weighed at day 52. The three tumor tissues of the RNAi group were smaller than those from the NC group (**P < 0.01).

To verify the positive role of TRIM47 on tumorigenicity *in vivo*, we performed xenograft tumor assays using A549 cells stably infected by scramble shRNA control or TRIM47-shRNA lentiviruses. As shown in Figure 5, we found that TRIM47 knockdown significantly inhibited the tumor growth rate in nude mice (*P* < 0.01). The weight of TRIM47-depleted tumors was less than that of control tumors. Collectively, our *in vitro* and *in vivo* observations suggest that TRIM47 functions as an oncogene in NSCLC.

### Identification of potential signaling pathways and processes

**Figure 6:**
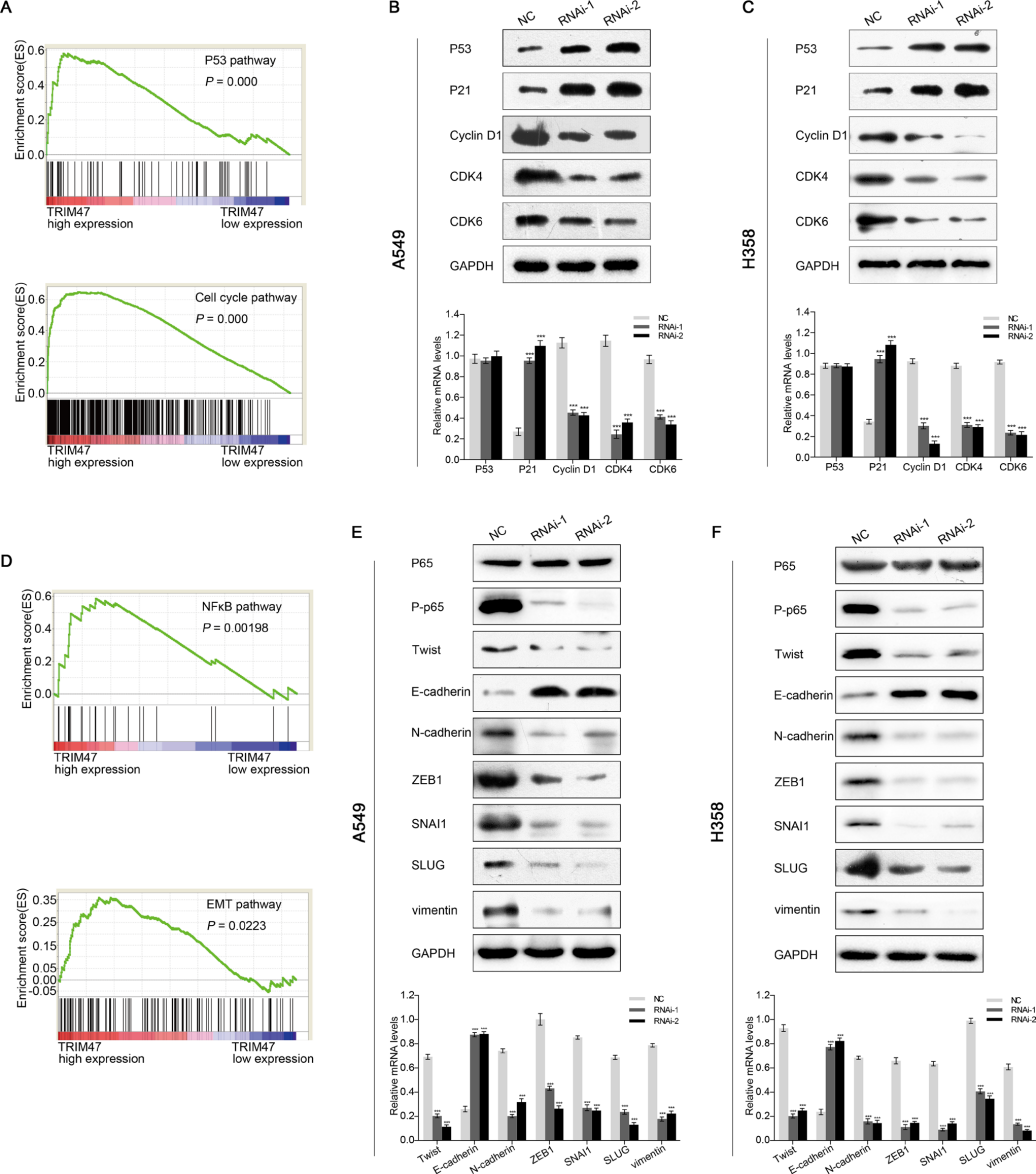
TRIM47 promotes NSCLC cell growth through p53-cell cycle and NFκB -EMT signaling pathway (**A**, **D**)Performance of GSEA based on TCGA dataset. High expression of TRIM47 correlated with p53-cell cycle (A) and NFκB-EMT (D) signaling pathway. (**B**, **C**) The expression of p53-cell cycle related genes. (**E**, **F**) The expression of NFκB-EMT related genes. Data were based on at least three independent experiments and are shown as the mean ± S.D. Representative images and quantitative results were shown (***P < 0.001).

As described above, how TRIM47 promotes proliferation and metastasis remained unknown. High throughput RNA-sequencing data of the NSCLC cohort from TCGA and GEO were used to perform GSEA to probe the TRIM47-associated pathways in an unbiased manner. The result showed that high expression of TRIM47 was related to p53-cell cycle and NFκB-EMT signaling pathway, which were associated with proliferation and metastasis (Figure 6A, 6D).

### P53-cell cycle pathway was associated with TRIM47 depletion-induced G1 phase arrest

The protein and mRNA levels of p53, p21, cyclin D1, CDK4, and CDK6, which are closely related to p53-cell cycle pathway, were detected by Western blot and real-time PCR. We found that the p53 protein level increased after TRIM47 knockdown (Figure 6B, 6C). Both the protein and mRNA level of p21 were up-regulated in TRIM47-shRNA lentivirus-infected A549 and H358 cells. Additionally, the protein and mRNA levels of cyclin D1, CDK4 and CDK6 were down-regulated (Figure 6B, 6C). However, no change in the mRNA levels of p53 was observed in TRIM47-shRNA lentivirus-transfected cells (Figure 6B, 6C). There may be a post-transcriptional modification of p53 by TRIM47. Overall, our observations indicate that TRIM47 depletion in NSCLC cells enhanced p53 expression and induced G1 phase arrest, thus inhibiting cell proliferation.

### NFκB-EMT pathway was associated with TRIM47 depletion-inhibited metastasis

To investigate the function of TRIM47 in metastasis, the expression levels of genes related to the NFκB-EMT pathway were evaluated. The protein expression level of P-p65 was down-regulated after TRIM47 knockdown, whereas the overexpression of TRIM47 did not affect the p65 protein amount (Figure 6E, 6F). Meanwhile, a higher expression of an epithelial marker (E-cadherin) and a reduced expression of mesenchymal markers (ZEB-1, N-cadherin, Twist, SNAI1, SLUG, vimentin) were evident in TRIM47-shRNA lentivirus-infected cells (Figure 6E, 6F). The above results indicate that TRIM47 exerts an activating effect on NFκB-EMT pathway, thereby promoting tumor metastasis in NSCLC.

## DISCUSSION

Overexpression of several TRIM family proteins has been implicated in some cancers [16–21]. However, the prognostic value, expression pattern and functional implication of TRIM47 in NSCLC have been poorly defined. In the present study, experimental evidence has been provided that TRIM47 is significantly overexpressed in NSCLC. High TRIM47 expression levels correlated with tumor differentiation, TNM stage, lymph node metastasis and tumor size (Table 1). Multivariable Cox regression analyses showed that overexpression of TRIM47 (*P* = 0.017) could be considered an independent prognostic factor for NSCLC (Supplementary Table 1). Several independent datasets from TCGA and GEO (Figure 1C–1F) also revealed that TRIM47 overexpression were associated with poor prognosis of patients with NSCLC. The ROC curves analysis demonstrated that the combination of TRIM47 and TNM stage was superior to TRIM47 or TNM stage alone for the prediction of patient survival (Figure 1J). Stated thus, TRIM47 is suggested as a potent biomarker for the early diagnosis and evaluation of the prognosis of patients with NSCLC.

Then we examined the association between TRIM47 and NSCLC by knockdown its expression in two cell lines. We found that TRIM47 depletion significantly inhibited cell proliferation (Figure 2), induced G0/G1 phase arrest in NSLCL cells (Figure 3), and inhibited cell migration and cell invasion (Figure 4). Moreover, TRIM47 silencing notably suppressed tumorigenicity in nude mice (Figure 5). These data demonstrated the association of TRIM47 with the carcinogenesis of NSCLC.

The exact pathway that TRIM47 may regulate in NSCLC remains unclear. GSEA was performed to elucidate how TRIM47 promotes proliferation and metastasis. We found that p53-cell cycle and NFκB-EMT pathways were positively associated with TRIM47 expression (Figure 6A, 6D).

Most TRIM proteins have ubiquitin E3 ligase activity and lead to the degradation of proteins [7]. TRIM24 and TRIM59 negatively regulate p53 through its E3 ligase activity [18, 22]. P21 is an important molecule downstream of p53, which negatively regulates the expression of cyclin D1 and CDK4/6 [23]. CDK4/6 can act to trigger early G1 entry from quiescence and facilitate G1 progression by cooperating with cyclin D1, thus promoting cell proliferation [24]. Here, we found that the p53 protein level increased after TRIM47 depletion. However, no change in the mRNA levels of p53 was observed. TRIM47 may bind to p53 and promote its ubiquitination. Both the protein and mRNA levels of p21 were up-regulated. However, the protein and mRNA levels of cyclin D1, CDK4 and CDK6 were down-regulated (Figure 6B, 6C). Our data suggested that TRIM47 promotes cell proliferation through down-regulating p53. However, further investigation is needed to explore the specific mechanism.

EMT is involved in tumor cell invasion and metastasis, which is critically required for the metastasis of NSCLC [25]. Nuclear factor-κB (NFκB) activation is suggested to stimulate the initiation and metastatic progression of cancer [26]. Activated NFκB induced the occurrence of EMT because there was an increase of ZEB-1 promoter activity, a specific reduction in E-cadherin expression and a gain of vimentin expression [27]. Our data demonstrated that the P-p65 protein level was down-regulated after TRIM47 knockdown. Increased expression of epithelial marker (E-cadherin) and decreased expression of mesenchymal markers (ZEB-1, N-cadherin, Twist, SNAI1, SLUG, and vimentin) were evident in TRIM47 silencing cells (Figure 6E, 6F). TRIM47 may induce the ubiquitination of IκB and activation of NFκB, thus promoting metastasis of NSCLC [28].

In summary, our data demonstrated that TRIM47 has an inhibitory action on p53 and its downstream molecules, thereby promoting tumor proliferation. Meanwhile, TRIM47 can activate the NFκB-EMT pathway, thus promoting tumor metastasis. TRIM47 overexpression may serve as a useful prognostic factor and a potential treatment target for NSCLC.

## MATERIALS AND METHODS

### Patients and tissue samples

In total, 90 patients with NSCLC were enrolled in our study from February 2006 to December 2006 at the Department of Thoracic Surgery, Shanghai General Hospital. Through reviewing medical records, we obtained clinicopathological variables, including diagnosis age, gender, tumor size and tumor differentiation. Forty-five fresh samples of NSCLC and paired normal adjacent tissues were frozen in liquid nitrogen within 10 minutes and stored at −80°C until quantitative real-time polymerase chain reaction was performed. Informed consent was obtained from patients and their families. This study was approved by the ethics committee of Shanghai General Hospital (Shanghai, China), Shanghai Jiao Tong University School of Medicine.

### Cell culture

According to ATCC protocols, NSCLC cell lines (A549, H460, H1299, SPC-A1, H292, and H358) and HEK293T cells (both purchased from the cell bank of Shanghai Biology Institute, Chinese Academy of Science) were cultured in Roswell Park Memorial Institute-1640 (Life Technologies, Carlsbad, CA, USA) and DMEM (Life Technologies), respectively, at 37°C in a humidified incubator under 5% CO2 conditions. Fetal bovine serum (10%) (FBS, Life Technologies) and 1% penicillin/streptomycin (Life Technologies) were supplemented into the culture media.

### Immunohistochemistry

To detect the TRIM47 expression level in NSCLC tissues and normal adjacent tissues, streptavidin peroxidase immunohistochemistry was used. The sections were incubated at 4°C overnight with rabbit anti-human TRIM47 antibody (1:100; HPA014933, Sigma) after deparaffinization and antigen retrieval. Then, the sections were incubated with a drop of biotin-labeled secondary antibody for 30 minutes at 37°C. We subsequently added a drop of horseradish peroxidase-conjugated streptomycin working solution to the sections and incubated them for 30 minutes at 37°C. Visualization was performed with 3,3′-diaminobenzidine (DAB), and sections were counterstained with hematoxylin.

TRIM47 expression was assessed with regard to the percentage of positive cells and the staining intensity. The staining intensity was scored as follows: 0 for no intensity, 1 for no or very weak staining, 2 for moderate intensity, and 3 for strong intensity. The percentage of positive cells was rated as follows: 0 for < 10%, 1 for 10% to < 25%, 2 for 25% to < 50%, and 3 for > 50%. Total histological score = staining intensity + percentage of positive cells. The patients could be classified into two groups: negative expression group (0–2) and positive expression group (3–6).

### RNA interference

Two shRNA sequences (TRIM47-Ri-1: 5′-CGCGTCCCCGCAGCTGTTTGGAACCAAATTCAAGAGATTTGGTTCCAAACAGCTGC -3′; TRIM47-Ri-2: 5′-CG CGTCCCCCCAGGGACTATTTCCTCAATTCAAGAGA TTGAGGAAATAGTCCCTGG-3′ ) and a non-specific scramble shRNA sequence were synthesized by Genepharm Technologies (Shanghai, China). The shRNA were inserted into pLVTHM vector (Addgene, Cambridge, MA, USA), then, the lentiviral packaging plasmid was transfected into HEK293T cells with Lipofectamine 2000 (Invitrogen, Carlsbad, CA, USA). Lentiviruses were collected at 48 hours or 72 hours after transfection, and we used the lentiviruses to infect A549 and H358 cells.

### Quantitative real-time PCR

Total RNA of specimens and cells harvested and washed with PBS were extracted with TRIzol reagent (Invitrogen). cDNA was then reverse transcribed with oligodT using M-MLV reverse transcriptase (Thermo Fisher Scientific, Rockford, IL, USA). Quantitative real-time PCR was performed with Maxima SYBR Green qPCR Master Mixes (Thermo Fisher Scientific) in an ABI 7300 system (Applied Biosystem, Foster City, CA, USA). The relative expression levels of genes of interest were determined using the 2−ΔΔCt method, GAPDH served as an internal control. The primers used are listed in Supplementary Table 3.

### Western blot analysis

RIPA lysis buffer (Thermo Fisher) with protease inhibitor (Sigma) was used to extract the total protein. The same amounts of total proteins were separated via SDS-PAGE gel and then transferred to polyvinylidene fluoride (PVDF, Millipore, Bedford, MA, USA) membranes. After blocking with 5% skim milk, the membranes were incubated with the primary antibodies[TRIM47 (Sigma); Twist, E-cadherin, N-cadherin, ZEB1, SNAI1, SLUG, and vimentin (Abcam); p53, P21, CDK4, CDK6, cyclin D1, p65, P-p65, and GAPDH (CST)] overnight at 4°C and then with the horseradish peroxidase-conjugated secondary antibody (Santa Cruz Biotechnology, CA, USA). Signals were detected with enhanced chemiluminescence system (Bio-Rad, Richmond, CA, USA).

### Cell viability assays

A549 and H358 cells were seeded in 96-well plates at a density of 1 × 10^4^ cells/well and then incubated for 24, 48, or 72 hours. Following the manufacturer's instructions, cell viability was assessed using a Cell Counting Kit-8 (Dojindo Lab, Kumamoto, Japan). A microplate reader (Bio-Rad) was used to measure the optical density values of each well at a wavelength of 450 nm.

### Cell cycle distribution assay

Treated and untreated cells were harvested by trypsinization, washed in PBS and fixed with ice-cold 70% ethanol at −20°C for at least 2 hours. After washing with PBS, cells were incubated with ribonuclease (Sigma) at 37°C for 15 min and then incubated with PI (0.05 mg/ml, Sigma) in the dark for 30 min at room temperature. Finally, a flow cytometer (BD Biosciences) was used to analyze DNA content.

### Migration and invasion assay

Tumor cell migration and invasion assays were performed using Boyden chambers (Coring Incorporated, NY, USA) containing polycarbonate filters (pore size, 8 μm). Cells transfected with the indicated virus were serum starved for 24 h and seeded in the upper chamber; then, medium containing 20% FBS was added in the lower chamber. After 24 h of incubation, cells on the upper side of the filter were completely removed. The remaining cells were fixed in 4% paraformaldehyde and stained with 0.05% crystal violet after washing with PBS. The cells on the lower surface of the filters were defined as migrating cells and counted under the microscope (Lake Success, NY, USA).

Invasion assays were also performed in Boyden chambers with polycarbonate filters coated with Matrigel (BD Biosciences) on the upper side. The rest was performed as migration assay.

### *In vivo* xenograft assay

The experimental procedures were approved by the Institutional Animal Care and Use Committee of Shanghai Jiao Tong University School of Medicine. Male BALB/c-nu mice (4–5 weeks old, 18–20 g) were purchased from Shanghai Laboratory Animal Company (SLAC, Shanghai). For each cell line, cell suspensions (2 × 10^6^ cells) in a total volume of 100 μl were injected subcutaneously into the right flanks of nude mice. Tumor length and width were measured and recorded every 4 days starting 2 weeks after inoculation. Tumor volume was calculated as 1/2×length×width^2^.

### Gene set enrichment analysis (GSEA)

The TCGA dataset of NSCLC was analyzed using GSEA2-2.2.2, as previously described [29]. The gene sets showing a false discovery rate (FDR) of 0.25, a well-established cutoff for the identification of biologically relevant gene, were considered enriched between the classes under comparison. The “c2.all.v5.0.symbols.gmt” from the Molecular Signatures Database–MSigDB (http://www.broad.mit.edu/gsea/msigdb/index.jsp) were used to run GSEA and 1000 permutations were used to calculate the *P-value*; the “permutation type” was set to “gene set”. All other parameters were set to default, except that the gene set should represent at least 15 genes.

### Data analysis

The statistical analyses were performed using SPSS version 16 software (Chicago, IL, USA), and graphical representations were generated with GraphPad Prism 5 software (San Diego, CA, USA). The data are presented as the means ± S.D., and Student's *t-test* was used to evaluate differences between groups. The relationship between the clinicopathological features and the expression of TRIM47 was evaluated by chi-square tests. Overall survival analyses were assessed by the Kaplan–Meier method with the log-rank test and Cox regression analysis. Receiver operating characteristic (ROC) analysis was used to calculated the sensitivity and specificity of TRIM47 as a diagnosis biomarker. *P* < 0.05 was regarded as significant.

## SUPPLEMENTARY MATERIALS FIGURES AND TABLES


