# Supplementary material for: TRIM47 overexpression is a poor prognostic factor and contributes to carcinogenesis in non-small cell lung carcinoma
Source: Oncotarget. 2017 Feb 8;8(14):22730–40. doi: 10.18632/oncotarget.15188 (PMC5410258; doi:10.18632/oncotarget.15188)
Supplement: Supplementary file 1 [file oncotarget-08-22730-s001.pdf]

TRIM47 overexpression is a poor prognostic factor and contributes to carcinogenesis in non-small cell lung carcinoma

Supplementary Materials

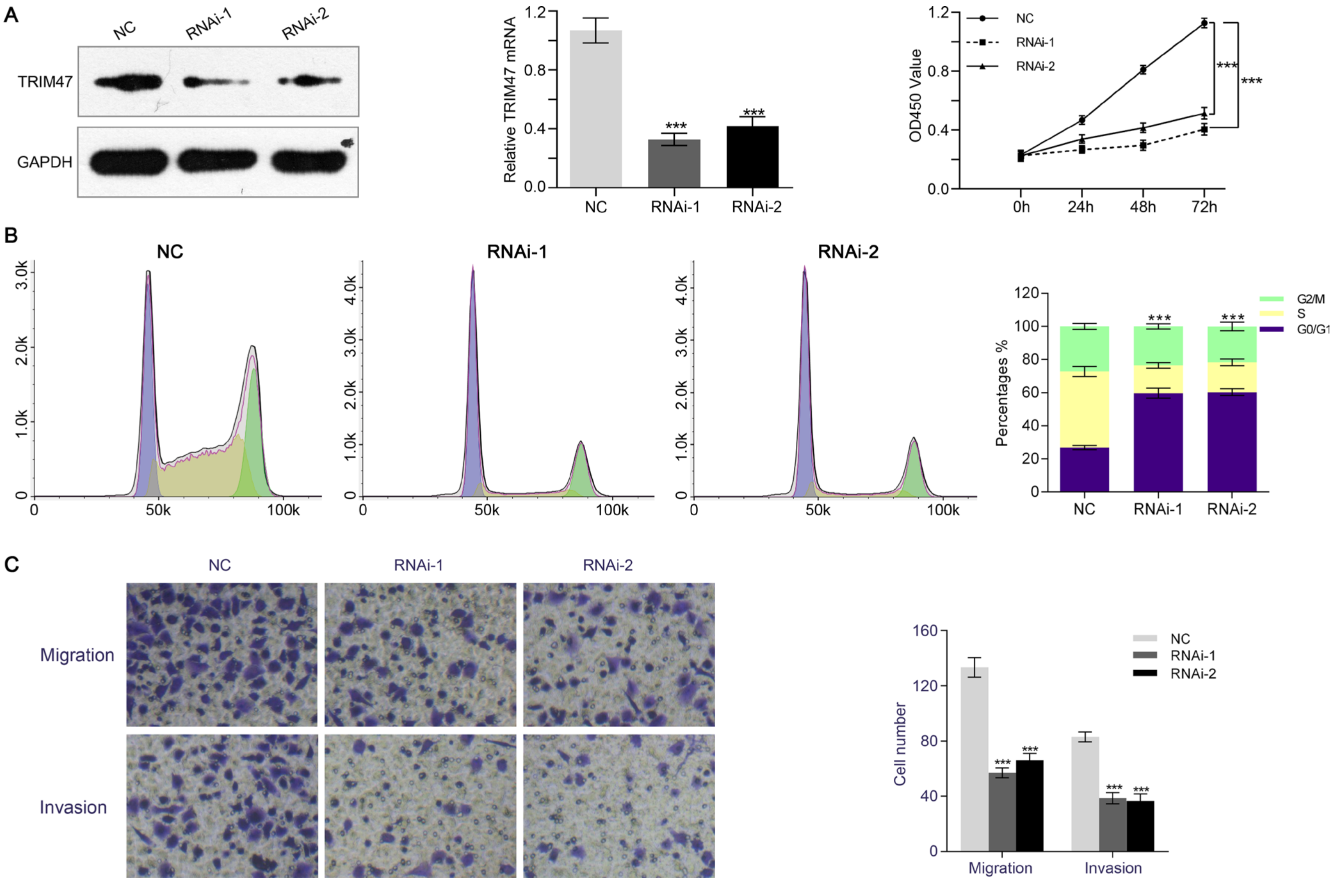

Supplementary Figure S1: TRIM47 depletion inhibited the proliferation, migration and invasion of H460 cells. (**A**) Expression of TRIM47 in H460 cells was analyzed by Western blot (left panel) and real-time PCR (middle panel). Cell proliferation (right panel) was detected 24, 48 and 72 hours after infection. (**B**) Silencing of TRIM47 induced G0/G1 arrest in H460 cells. (**C**) Knockdown of TRIM47 inhibited migration and invasion of H460 cells (***P < 0.001).

Supplementary Table 1: Univariate and multivariable Cox regression analysis of the association between clinicopathological features and overall survival

Characteristics	HR (95% CI)	*P*
**Univariate analysis**		
Age	1.170 (0.677–2.022)	0.574
Gender	1.282 (0.754–2.181)	0.360
Histological type	1.373 (0.836–2.054)	0.211
Tumor differentiation	1.400 (0.850–2.305)	0.186
TNM stage	8.003 (4.404–14.541)	< 0.001***
Lymph node metastasis	7.297 (3.977–13.387)	< 0.001***
Tumor size	1.457 (0.883–2.404)	0.141
TRIM47 expression	2.083 (1.223–3.548)	0.007**
**Multivariate analysis**		
TNM stage	3.131 (1.443–6.796)	0.004**
Lymph node metastasis	3.784 (1.672–8.563)	0.001**
TRIM47 expression	1.946 (1.124–3.369)	0.017*

Note: *P* values are from chi-square test and were significant at < 0.05. *$P < 0.05$, **$P < 0.01$, ***$P < 0.001$.

Supplementary Table 2: Relationship between TRIM47 mRNA expression and clinicopathological parameters in NSCLC patients

Variables	No. of patients	TRIM47 expression		*P*
		High	Low	
Age				0.833
< 58	15	8	7	
≥ 58	30	15	15	
Gender				0.750
Male	29	15	14	
Female	16	8	8	
Histological type				0.641
Squamous cell carcinoma	20	11	9	
Adenocarcinoma	25	12	13	
Tumor differentiation				0.010*
Well	26	9	17	
Poor	19	14	5	
TNM stage				0.003**
I + II	27	9	18	
III + IV	18	14	4	
Lymph node metastasis				0.002**
No	22	6	16	
Yes	23	17	6	
Tumor size				0.025*
< 3 cm	19	6	13	
≥ 3 cm	26	17	9	

Note: *P* values are from chi-square test and were significant at < 0.05. **P* < 0.05, ***P* < 0.01.

Supplementary Table 3: Primers for real-time RT-PCR

	Primer sequences (5′→3′)
TRIM47	5′ GTCCAAAGTCCTGAGCGCC 3′
	5′ GCTACGGCTGCACTCTTGAT 3′
P53	5′ AACGGTACTCCGCCACC 3′
	5′ CGTGTCACCGTCGTGGA 3′
P21	5′ TGGAGACTCTCAGGGTCGAAA 3′
	5′ GGCGTTTGGAGTGGTAGAAATC 3′
Cyclin D1	5′ TGGAGGTCTGCGAGGAACA 3′
	5′ TTCATCTTAGAGGCCACGAACA 3′
CDK4	5′ ACTGGCCTCGAGATGTATCC 3′
	5′ TGCTGCAGAGCTCGAAAGGC 3′
CDK6	5′ CGTGGTCAGGTTGTTTGATGTG 3′
	5′ ACTCGGTGTGAATGAAGAAAGTCC 3′
Twist	5′ GCAGGACGTGTCCAGCTC 3′
	5′ CTGGCTCTTCCTCGCTGTT 3′
E-cadherin	5′ CGGGAATGCAGTTGAGGATC 3′
	5′ AGGATGGTGTAAGCGATGGC 3′
N-cadherin	5′ CACTGCTCAGGACCCAGAT 3′
	5′ TAAGCCGAGTGATGGTCC 3′
ZEB1	5′ AGCAGTGAAAGAGAAGGGAATGC 3′
	5′ GGTCCTCTTCAGGTGCCTCAG 3′
SNAI1	5′ ACTGCAACAAGGAATACCTCAG 3′
	5′ GCACTGGTACTTCTTGACATCTG 3′
SLUG	5′ ATCTGCGGCAAGGCGTTTTCCA 3′
	5′ GAGCCCTCAGATTTGACCTGTC 3′
Vimentin	5′ GACAATGCGTCTCTGGCACGTCTT3′
	5′ TCCTCCGCCTCCTGCAGGTTCTT3′
GAPDH	5′CACCCACTCCTCCACCTTTG3′
	5′CCACCACCCTGTTGCTGTAG3′
